# Advanced therapy treatment patterns in moderate-to-severe ulcerative colitis: a long-term retrospective claims analysis

**DOI:** 10.1093/crocol/otag035

**Published:** 2026-04-22

**Authors:** Parambir S Dulai, Christopher J Rabbat, Ian Nason, Yechu Hua, Sydney Ng, Yixin Yang, Noam Kirson, Jennifer T Fine

**Affiliations:** Division of Gastroenterology and Hepatology, Feinberg School of Medicine, Northwestern University, Chicago, IL, United States; Abivax, Boston, MA, United States; Analysis Group, Inc., New York, NY, United States; Analysis Group, Inc., Boston, MA, United States; Analysis Group, Inc., Los Angeles, CA, United States; Analysis Group, Inc., Menlo Park, CA, United States; Analysis Group, Inc., Boston, MA, United States; Abivax, Boston, MA, United States

**Keywords:** ulcerative colitis, treatment patterns, advanced therapies, long-term follow-up, real-world evidence

## Abstract

**Background:**

There has been significant evolution in advanced therapy (AT) options for moderate-to-severe ulcerative colitis (UC). We assessed shifts in treatment patterns over time using a large claims database with long-term follow-up.

**Methods:**

This retrospective study utilized the IQVIA PharMetrics Plus claims database (2012-2023) to analyze adults in the United States with UC who initiated an AT. Patients had ≥3 years of continuous follow-up after AT initiation and no evidence of other autoimmune diseases during the six-month baseline period. Treatment patterns including persistence, switching, and/or discontinuation were summarized. Analyses were stratified by year of AT initiation: 2012-2014, 2015-2016, 2017-2018, and 2019-2020.

**Results:**

6726 patients with a mean follow-up of 61.5 months and treatment duration of 22.9 months (SD: 14.2) were included. Vedolizumab usage increased over time in parallel with a reduction in anti-TNF therapy. Around 27%-34% of patients had dose escalation and 40% switched ATs at some point during follow-up. AT treatment persistence rates at 3 years of follow-up were <40% across all time periods, and <10% when limiting to patients who did not require dose escalation and/or steroids. Approximately 57% of patients in routine practice discontinued AT and did not start a subsequent AT.

**Conclusion:**

Despite the availability of multiple new AT options for UC, rates of steroid-free persistence without the need for dose escalation were low. High rates of treatment discontinuation across lines of therapy highlight a need for durable treatment options.

Key messages **What is already known?** 

- Prior studies using administrative claims data have identified high rates of AT discontinuation and switching but have generally been limited by short follow-up periods, thereby limiting the ability to truly understand durability of treatments.

**What is new here?** 

- In this long-term (≥3 years) real-world study, over half of UC patients discontinued their AT, and fewer than 20% remained on their initial AT without dose escalation or steroid use.

**How can this study help patient care?** 

- This study can help patient care by underscoring the unmet need for durable, steroid-free remission in UC despite an expanding number of approved therapies.

## Introduction

Ulcerative colitis (UC) affects over 1 million Americans and 2 million Europeans among an estimated 5 million adults globally, with significant impacts on quality of life and healthcare resource utilization.^1–4^ The primary therapeutic goal for patients with moderate-to-severe UC is to induce and maintain long-term steroid-free disease remission. Gastroenterological societal guidelines strongly recommend the use of advanced therapies (AT), such as tumor necrosis factor antagonists (anti-TNFs; infliximab, adalimumab, and golimumab), or more recently approved therapies including ustekinumab, vedolizumab, or Janus Kinase inhibitors (JAKs; tofacitinib, upadacitinib), to achieve these treatment goals.^5–7^

Prior work has demonstrated that despite the growing number of therapeutic options, there is persistent unmet need in utilization of these AT for patients with moderate-to-severe UC.^8^ Furthermore, once AT is started, a substantial proportion of these patients still require chronic steroid use with high rates of treatment failure.^9–11^ This results in persistent disability from the condition and the need to frequently cycle between multiple ATs over time.^10^^,^^12^ Although these prior studies have been helpful in identifying gaps, they have been limited by relatively short follow-up periods for patients after starting AT, inability to follow individual patients through the various cycles of AT switching, and lack of granularity on dose escalation and/or concomitant steroid use over time. A broadly encompassing study is needed to define current landscapes and persistent gaps for UC in the United States.

This study aims to provide an updated characterization of the real-world treatment landscape for AT in UC by leveraging large administrative claims data to examine long-term (≥3 years) treatment patterns in UC patients initiating AT over a 10-year period.

## Materials and methods

### Data source and study design

This was a retrospective analysis using the IQVIA PharMetrics Plus claims database, comprising medical and pharmacy claims from U.S. commercial health plans (2012-2023). IQVIA PharMetrics Plus is a comprehensive database that contains longitudinal data on enrollment and adjudicated medical and pharmacy claims for approximately 215 million enrollees in the US since 2006. The database contains a diverse representation of geographic areas, employers, providers, therapeutic areas, and payers across US. Enrollment data allowed us to ensure continuous coverage in medical and pharmacy benefit. Unlike other claims datasets, which often lack complete visibility across payers, have limited longitudinal tracking, or exclude certain provider types, the IQVIA PharMetrics Plus dataset offers comprehensive, adjudicated medical and pharmacy claims from a broad network of commercial health plans, enabling robust longitudinal follow-up, diverse population representation, and detailed information on diagnoses, procedures, and prescriptions plans, facilitating comprehensive capture of each patient’s full treatment journey.

### Patient selection

Adult patients (18+ years) with claims for AT for UC between January 1, 2012 and December 31, 2020 were identified (Appendix Figure S1). The index date was defined as the date of first AT initiation during the identification period. During the 6-month baseline period, patients were required to have continuous eligibility in both medical and pharmacy claims and no claims for Crohn’s disease, other specific autoimmune diseases, malignancies, or use of an AT (Appendix Table S1). Patients were also required to have at least 2 claims with a diagnosis of UC (ICD-9-CM: 556; ICD-10-CM: K51) on separate dates in the 6 months prior to and/or at the initiation of a therapy for moderate-to-severe UC (Appendix Table S2) and continuous eligibility for at least 36 months after the index date.

For certain analyses, patients were stratified into four index year cohorts: 2012-2014, 2015-2016, 2017-2018, and 2019-2020 to identify whether observed treatment patterns were influenced by temporal evolutions in treatment options with the introduction of vedolizumab to the market in 2014, tofacitinib in 2018, and ustekinumab in 2019.

### Study measures

Patient demographic and baseline characteristics included age at index date, Charlson Comorbidity Index (CCI) score, individual comorbidities related to UC, and prior treatment history. Individual comorbidities with fewer than 11 patients were not included to protect patient privacy. The follow-up period was defined as time between the first day of supply of an AT to the end of continuous eligibility or the end of data availability, whichever was earlier. Outcomes measured during the follow-up period included duration of treatment, dose escalation, complete discontinuation, switching, restarting, and persistence.

Treatment sequences by individual treatment and by treatment class were summarized up until the fourth line of therapy. Lines of therapy were defined by the sequential order in which a patient received an individual AT. For example, a patient initially prescribed adalimumab, followed by vedolizumab at a later date, would be categorized as receiving first-line (1L) adalimumab and second-line (2L) vedolizumab. The first through fourth lines of therapy are referred to as 1L, 2L, 3L, and 4L, respectively.

Duration of treatment was defined as the time from the initiation of an AT, measured as a pharmacy fill, to the last day of supply of the last claim for the AT. Treatment persistence was defined as remaining on the index AT and was examined at one, two, and three years after the index date. A more restrictive measure of treatment persistence was also examined, that further restricted to patients remaining on the index AT without dose escalation or glucocorticoid (GC) use as this is more reflective of true treatment persistence and likely disease remission without the need to modify dosing strategies or use steroids for flares during the maintenance period. GC use after initiation of index AT was defined as any use of prednisone or budesonide after initiating index AT.

Dose escalation was defined as a dose increase during the maintenance phase (ie, the period following the U.S. Food and Drug Administration [FDA]-approved dosing schedule for induction, starting after the first observed claim of an AT). Dose escalations were defined as treatment interval shortening for infusions, or doses that were higher than a drug-specific threshold set relative to the recommended standard maintenance dose for oral medications. Intervals and thresholds varied by each treatment and were informed by both the FDA-approved label and clinical input. A more detailed description of the individual dose escalation thresholds is provided in Appendix Table S3.

Complete discontinuation was identified in two steps. First, we examined whether a patient had a gap in therapy after a fill for an AT greater than an allowable gap. The allowable gap was defined for each of the therapies as equal to the days of supply of a typical maintenance regimen plus 30 days, then rounded to the nearest 45, 60, or 90 days.^10^^,^^13^ For infusion treatments, the days of supply was assumed as the interval length of the maintenance regimen as included in the FDA-approved label. The last day of supply before discontinuation was defined as the discontinuation date. If the time between a patient’s last day of supply and the end of follow-up was shorter than the allowable gap, the patient was not classified as discontinued. Among patients with discontinuation, patients who had a claim for the same AT after having a treatment gap greater than the allowable gap and did not switch treatments were considered to have restarted the same treatment. Second, patients were classified as having completely discontinued if they had at least 1 year of follow-up after a discontinuation. This helped to ensure that the observed discontinuation was not due to the patient leaving the database given they had at least 1 full year of follow-up with AT use post-discontinuation. Censored discontinuation was defined as having less than 1 year of follow-up after a discontinuation. Switching was defined as having a claim for a different AT after initiation of the previous AT.

### Statistical analysis

Patient demographic and baseline characteristics were described for the overall population and for subgroups of patients who did not discontinue index AT, discontinued index AT with <1 year of post-treatment follow-up, and discontinued index AT with ≥1 year post-treatment follow up. All patient demographics, baseline characteristics, treatment patterns and outcomes were summarized using mean (± standard deviation [SD]) and median for continuous variables and frequency (proportion) for categorical variables. Sankey plots were used to examine treatment patterns by line of treatment. Analyses were performed using SAS 9.4 (SAS Institute, Inc, Cary, NC) and R (version 4.2.3).

## Results

### Sample selection

A total of 91 422 patients in the IQVIA PharMetrics Plus database had claims for AT indicated for UC. After applying additional selection criteria, a final analytic cohort of 6726 patients was identified for inclusion in the study. A sample selection flowchart is available in (Appendix Figure S1).

### Baseline characteristics

Baseline patient demographic and clinical characteristics by discontinuation status are shown in Table 1. The mean age of patients at index date was 38.9 years (standard deviation [SD]: 14.5 years). Most patients were male (53.3%) and had prior use of treatments for UC including GC (79.9%) and oral 5-aminosalycylic acid (75.0%). For the study cohort, the mean length of follow-up was 61.5 months (SD: 21.3 months). The mean duration of treatment was 22.9 months (SD: 14.2 months).

**Table 1:** Patient baseline characteristics.

			Discontinued Index AT	
	Total	Not Discontinued	Discontinued	Discontinued
			with < 1 year follow up	with ≥ 1 year follow up
	*N* = 6726	*N* = 2979	*N* = 780	*N* = 2967
**Demographics**				
** Age at index (years)**				
** Mean ± SD**	38.9 ± 14.5	38.6 ± 14.8	38.3 ± 14.1	39.4 ± 14.3
** Sex**				
** Female**	3142 (46.7%)	1414 (47.5%)	355 (45.5%)	1373 (46.3%)
** Male**	3584 (53.3%)	1565 (52.5%)	425 (54.5%)	1594 (53.7%)
**Clinical characteristics**				
** Time from first observed UC diagnosis to index date (month)**				
** Mean ± SD**	3.6 ± 1.7	3.6 ± 1.7	3.6 ± 1.7	3.6 ± 1.8
**Charlson Comorbidity Index, *n* (%)**				
** CCI composite score**				
** Mean ± SD**	0.2 ± 0.6	0.2 ± 0.6	0.2 ± 0.6	0.2 ± 0.7
** Chronic pulmonary disease**	550 (8.2%)	259 (8.7%)	56 (7.2%)	235 (7.9%)
** Mild liver disease**	346 (5.1%)	162 (5.4%)	35 (4.5%)	149 (5.0%)
** Diabetes without chronic complications**	329 (4.9%)	143 (4.8%)	32 (4.1%)	154 (5.2%)
**Comorbidities related to UC, *n* (%)**				
** Diarrhea**	3395 (50.5%)	1579 (53.0%)	354 (45.4%)	1462 (49.3%)
** Anemia**	1108 (16.5%)	515 (17.3%)	101 (12.9%)	492 (16.6%)
** Hypertension**	1009 (15.0%)	451 (15.1%)	109 (14.0%)	449 (15.1%)
** Obesity**	944 (14.0%)	449 (15.1%)	124 (15.9%)	371 (12.5%)
** Anxiety**	884 (13.1%)	400 (13.4%)	99 (12.7%)	385 (13.0%)
** Fatigue**	836 (12.4%)	374 (12.6%)	73 (9.4%)	389 (13.1%)
** Depression**	698 (10.4%)	317 (10.6%)	72 (9.2%)	309 (10.4%)
** Infections**	432 (6.4%)	225 (7.6%)	25 (3.2%)	182 (6.1%)
** Malnutrition**	293 (4.4%)	138 (4.6%)	20 (2.6%)	135 (4.6%)
**Prior UC treatments, *n* (%)**				
** Any prior use of UC treatments**	6192 (92.1%)	2801 (94.0%)	705 (90.4%)	2686 (90.5%)
**Glucocorticoids**				
** Any prior use of glucocorticoids**	5376 (79.9%)	2481 (83.3%)	586 (75.1%)	2309 (77.8%)
** Prednisone**	4505 (67.0%)	2096 (70.4%)	481 (61.7%)	1928 (65.0%)
** Budesonide**	1674 (24.9%)	771 (25.9%)	188 (24.1%)	715 (24.1%)
** Hydrocortisone**	921 (13.7%)	399 (13.4%)	87 (11.2%)	435 (14.7%)
** Methylprednisolone**	706 (10.5%)	319 (10.7%)	68 (8.7%)	319 (10.8%)
**Oral 5-ASA**				
** Any prior use of oral 5-ASA**	5045 (75.0%)	2310 (77.5%)	577 (74.0%)	2158 (72.7%)
** Mesalamine**	4590 (68.2%)	2112 (70.9%)	524 (67.2%)	1954 (65.9%)
** Balsalazide**	507 (7.5%)	218 (7.3%)	57 (7.3%)	232 (7.8%)
** Sulfasalazine**	342 (5.1%)	158 (5.3%)	44 (5.6%)	140 (4.7%)
**Immunomodulators**				
** Any prior use of immunomodulators**	1220 (18.1%)	487 (16.3%)	143 (18.3%)	590 (19.9%)
** Azathioprine**	1064 (15.8%)	418 (14.0%)	127 (16.3%)	519 (17.5%)
** Mercaptopurine**	579 (8.6%)	235 (7.9%)	68 (8.7%)	276 (9.3%)
** Methotrexate**	121 (1.8%)	50 (1.7%)	12 (1.5%)	59 (2.0%)

Abbreviations: 5-ASA, 5-aminosalycylic acid; CCI, Charlson Comorbidity Index; HIV/AIDS, human immunodeficiency virus/acquired immunodeficiency syndrome; SD, standard deviation; UC, ulcerative colitis.

(1) The index date was defined as the date of treatment initiation for a patient’s first treatment for moderate to severe UC.

(2) The baseline period was defined as the 6 months prior to the index date.

(3) The UC diagnosis was observed during the baseline period.

(4) The IQVIA data provides only the year of birth (YOB), so July 1st of the birth year was used to impute the date of birth (DOB). Age at the index date was then calculated as the difference between the index date and the imputed DOB.

(5) Some conditions may be under-reported in claims and therefore prevalence of UC-related comorbidities may be underestimated.

(6) For comorbidities included in the CCI, a total of 0 (0.0%) patients had claims with a diagnosis of any malignancy, including lymphoma and leukemia, except malignant neoplasm of skin, or metastatic solid tumor.

(7) For UC-related comorbidities, a total of 0 (0.0%) patients had claims with a diagnosis of smoking.

(8) A total of 0 (0.0%) patients had claims for treatment with Beclomethasone, Sirolimus, Thalidomide, or Thioguanine during the baseline period.

(9) Variables with cell sizes < 11 were suppressed to protect privacy and maintain compliance with data reporting guidelines. The comorbidities suppressed include peripheral vascular disease, renal disease, peptic ulcer disease, congestive heart failure, diabetes with chronic complications, myocardial infarction, cerebrovascular disease, rheumatic disease, moderate to severe liver disease, hemiplegia or paraplegia, HIV/AIDS, and dementia. Prior UC treatments suppressed include prednisolone, dexamethasone, olsalazine, tacrolimus, mycophenolate, and cyclosporine.

### Overall treatment patterns

**Figure 1:**
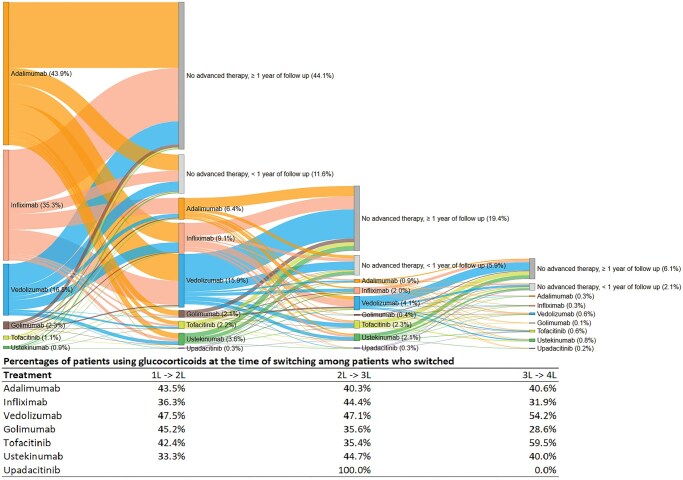
Sankey diagram.

Sankey diagrams (Figure 1) show the flow of patients through lines of therapy. Most patients initially received adalimumab (42.9%), infliximab (35.3%) or vedolizumab (16.5%) as their 1L AT. Around 40% switched therapies at least once and around 60% experienced discontinuation at some point during the follow-up period. Vedolizumab was the most common 2L AT. In 3L and 4L, there was greater variation in AT use, with ustekinumab being the most frequent. About 40% of patients had a GC (prednisone or budesonide) prescription at the time of switching therapies, with usage of GC increasing in later lines.

### Treatment patterns by index year cohorts

Baseline patient demographic and clinical characteristics by index year cohorts (2012-2014, 2015-2016, 2017-2018, and 2019-2020) are shown in Appendix Table S4. The prevalence of certain comorbidities such as obesity (7.9% to 17.0%) and anxiety (9.4% to 15.5%) increased over time, while the use of immunomodulators (24.9% to 13.2%) and GC (83.6% to 78.1%) declined from the 2012-2014 cohort to the 2019-2020 cohort.

**Figure 2:**
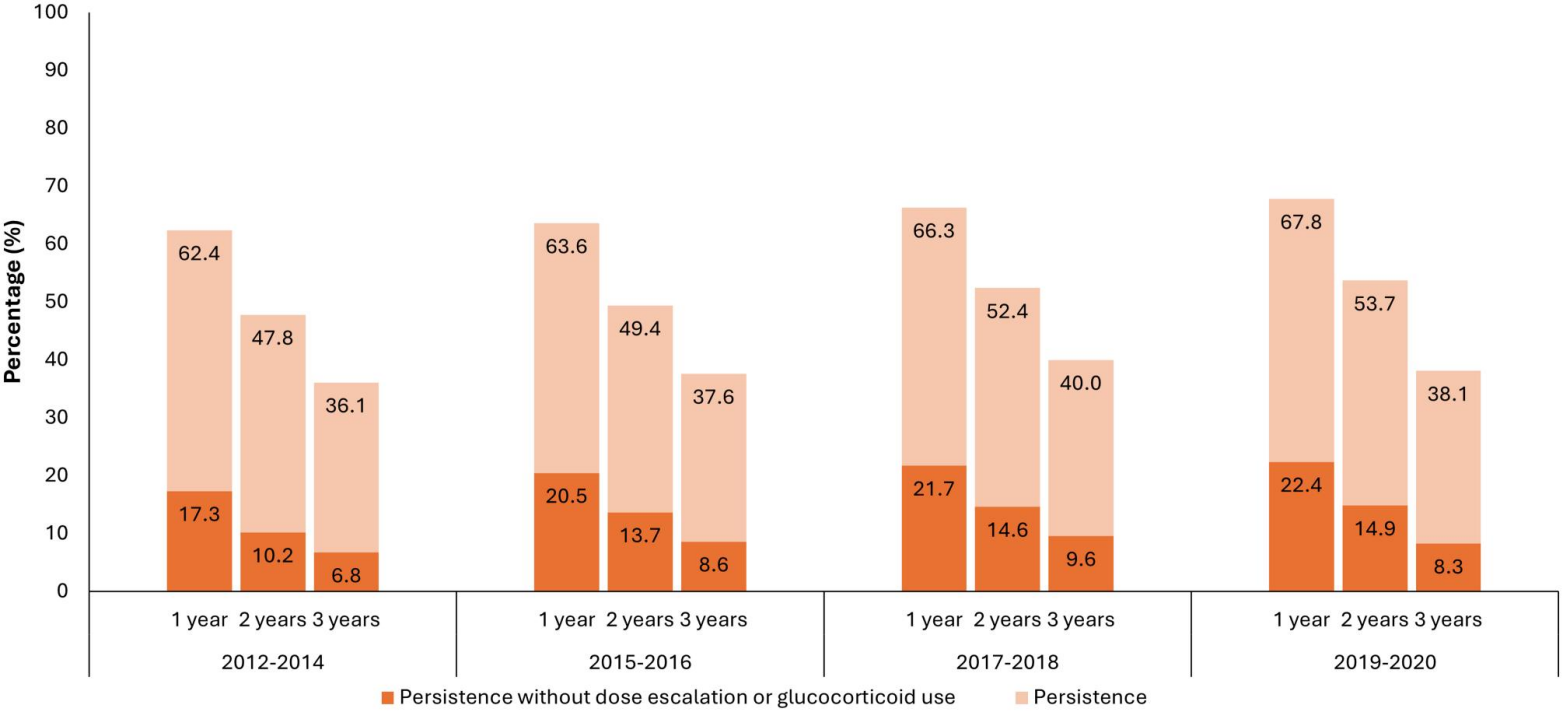
1L treatment persistence with and without dose escalation or GC use.

Table 2 presents the treatment patterns over time by index year. The proportion of patients using infliximab for 1L therapy fell overtime (49.6% to 26.4%), while the use of vedolizumab increased markedly (0.5% to 29.6%). At 3 years of follow-up, the proportion of patients remaining on their 1L AT was less than or equal to 40% across all index year cohorts, and the proportion of patients remaining on their 1L AT without the need for dose escalation and/or GC was less than 10% across all index year cohorts (Figure 2).

**Table 2:** 1L treatment patterns by index year.

	Index year of advanced therapy			
	2012-2014	2015-2016	2017-2018	2019-2020
**Patients with advanced therapy at index, %**				
** Infliximab**	49.6%	38.0%	35.0%	26.4%
** Adalimumab**	46.3%	47.3%	46.7%	38.0%
** Golimumab**	3.7%	4.4%	1.8%	0.5%
** Vedolizumab**	0.5%	10.3%	15.8%	29.6%
** Tofacitinib**	–	–	0.6%	2.9%
** Ustekinumab**	–	–	0.1%	2.7%
**Duration of treatment (months), mean ± SD**				
** Infliximab**	23.6 ± 14.1	24.2 ± 14.1	24.2 ± 14.2	24.2 ± 14
** Adalimumab**	20.3 ± 14.5	20.5 ± 14.4	20.9 ± 14.5	21.2 ± 14.3
** Golimumab**	16.3 ± 13.8	17.2 ± 14.5	23.8 ± 12.8	29.5 ± 12.2
** Vedolizumab**	25.1 ± 15.8	25.8 ± 13.1	27.3 ± 12.6	25.8 ± 13
** Tofacitinib**	–	–	18.2 ± 15.1	21.7 ± 15.3
** Ustekinumab**	–	–	–	26.2 ± 12.7
**Patients remaining on index AT, %**				
** At 12 months**	62.4%	63.6%	66.3%	67.8%
** At 18 months**	54.1%	55.7%	58.0%	59.8%
** At 24 months**	47.8%	49.4%	52.4%	53.7%
** At 30 months**	43.0%	45.8%	47.8%	49.1%
** At 36 months**	36.1%	37.6%	40.0%	38.1%
**Patients remaining on index AT without dose escalation or GC use, %**				
** At 12 months**	14.3%	18.1%	18.3%	19.4%
** At 24 months**	9.9%	13.0%	13.7%	14.2%
** At 36 months**	6.8%	8.6%	9.5%	8.2%
**Patients with >1 year of follow-up after discontinuation of 1L treatment, %**	55.8%	51.3%	50.4%	27.8%
**Patients with a treatment switch, %**	33.6%	38.9%	40.6%	42.3%
**Patients using GC after initiation of index AT, %**	35.3%	29.9%	24.4%	13.5%

Abbreviations: AT, advanced therapy; GC, glucocorticoid; L, line; SD, standard deviation.

Dose escalation was defined as an increase in AT dose or a shortening of prescription intervals, based on drug label indications and clinical input. GC use was defined as filling a prescription for prednisone or budesonide.

### Complete discontinuation

Among the 6726 patients who initiated an AT, 3747 participants (55.7%) discontinued their index AT without initiating a subsequent AT during the follow-up period (2967/3747 participants (79.2%) complete discontinuation with > 1 year of follow-up after discontinuation). (Appendix Table S5). Discontinuation rates varied across treatments and lines of therapy, with vedolizumab observed to have the highest discontinuation (59.1%) rates when used as a 1L agent (Figure 3A). Duration of treatment decreased (Figure 4) and discontinuation rates increased in later line of therapies (64.0% in 2L; 68.0% in 3L; 63.4% in 4L). (Figure 4; Appendix Table S5). GCs were used following 1-, 2-, 3-, and 4L therapy in 42.9%, 50.1%, 57.1%, and 51.6% of patients, respectively. (Appendix Table S5).

**Figure 3:**
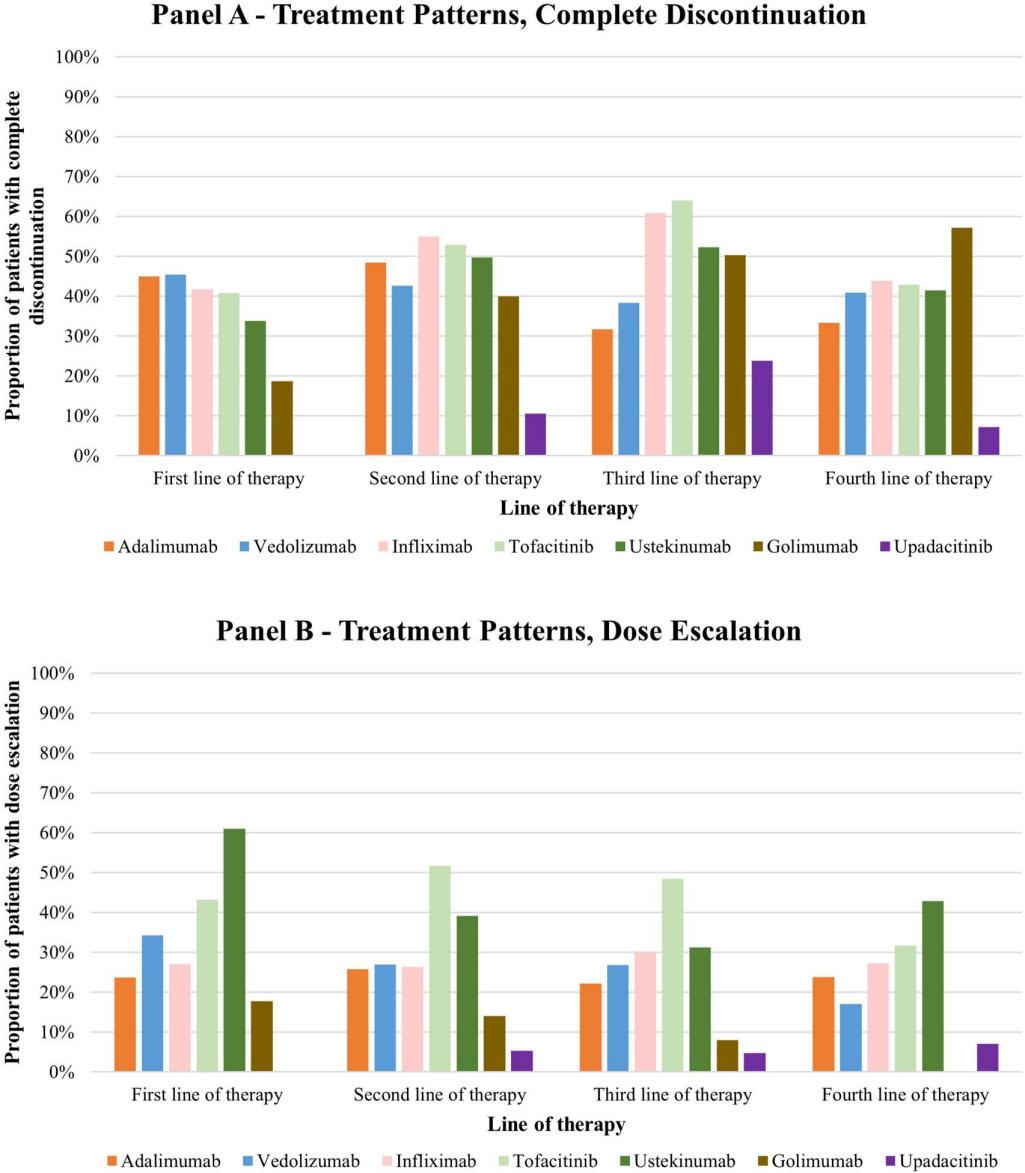
Treatment patterns. (A) Complete discontinuation. (B) Dose escalation. (C) Switching. (D) Restarting. *Notes*: (1) Dose escalation events included increased average daily doses due to increases in doses in each treatment interval or interval shortening. Thresholds varied by each treatment and were informed by both FDA USPIs and clinical input. (2) Treatment switching was defined as having a claim for a different advanced therapy after initiation of the previous advanced therapy. (3) Treatment restarting was defined as having a claim for the same advanced therapy after having a treatment gap greater than the allowable gap and did not include patients who switched treatments. The allowable gap was defined for each of the therapies as equal to the interval length of the maintenance regimen plus 30 days, then rounded to 45, 60, or 90 days. (4) Discontinuation was defined as having a treatment discontinuation on an advanced therapy without having a claim for a new advanced therapy during the remainder of the follow-up time, considering an allowable gap. Complete discontinuation was defined as having a treatment discontinuation and at least 1 year of follow-up.

**Figure 3:**
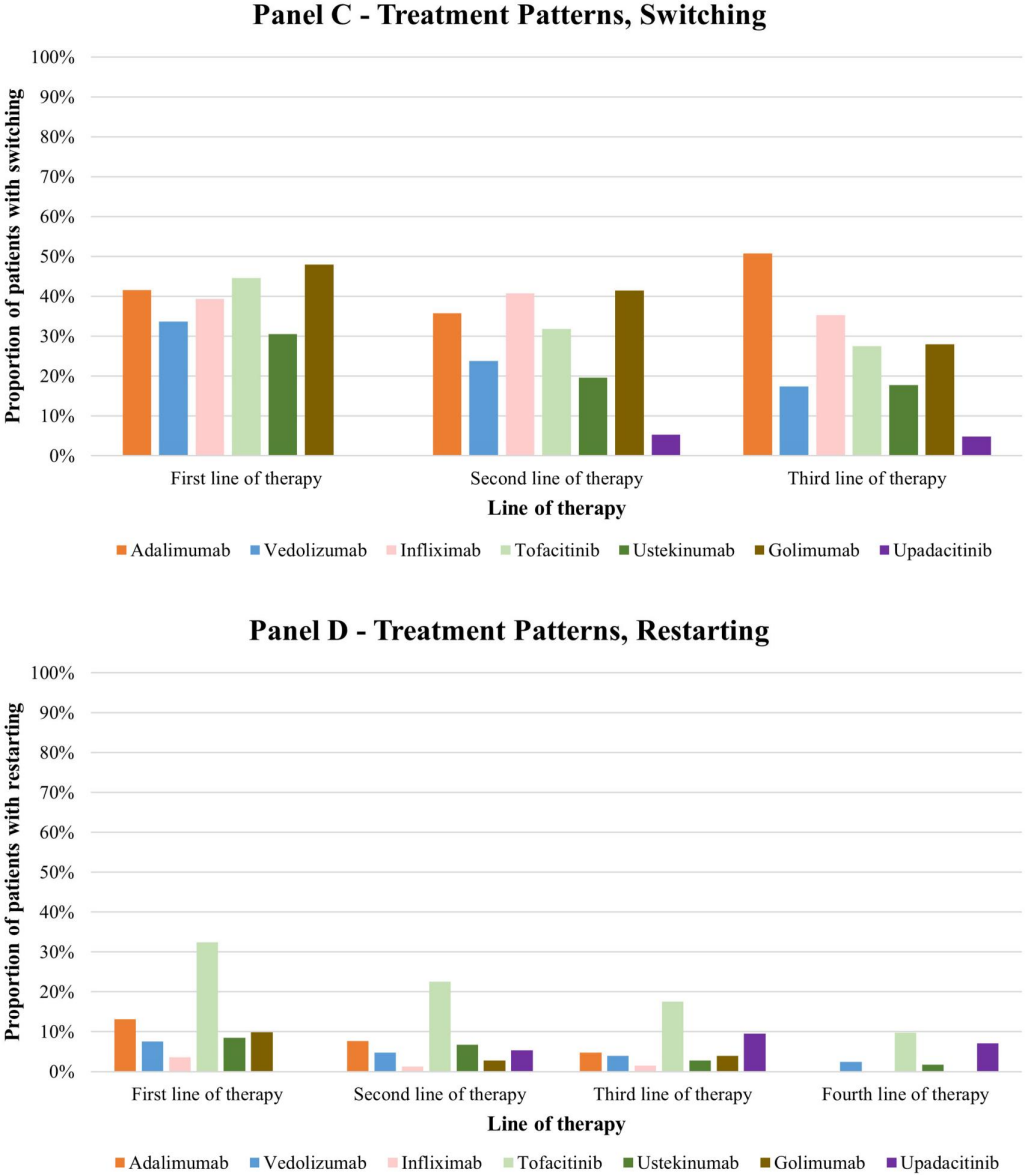
Continued.

**Figure 4:**
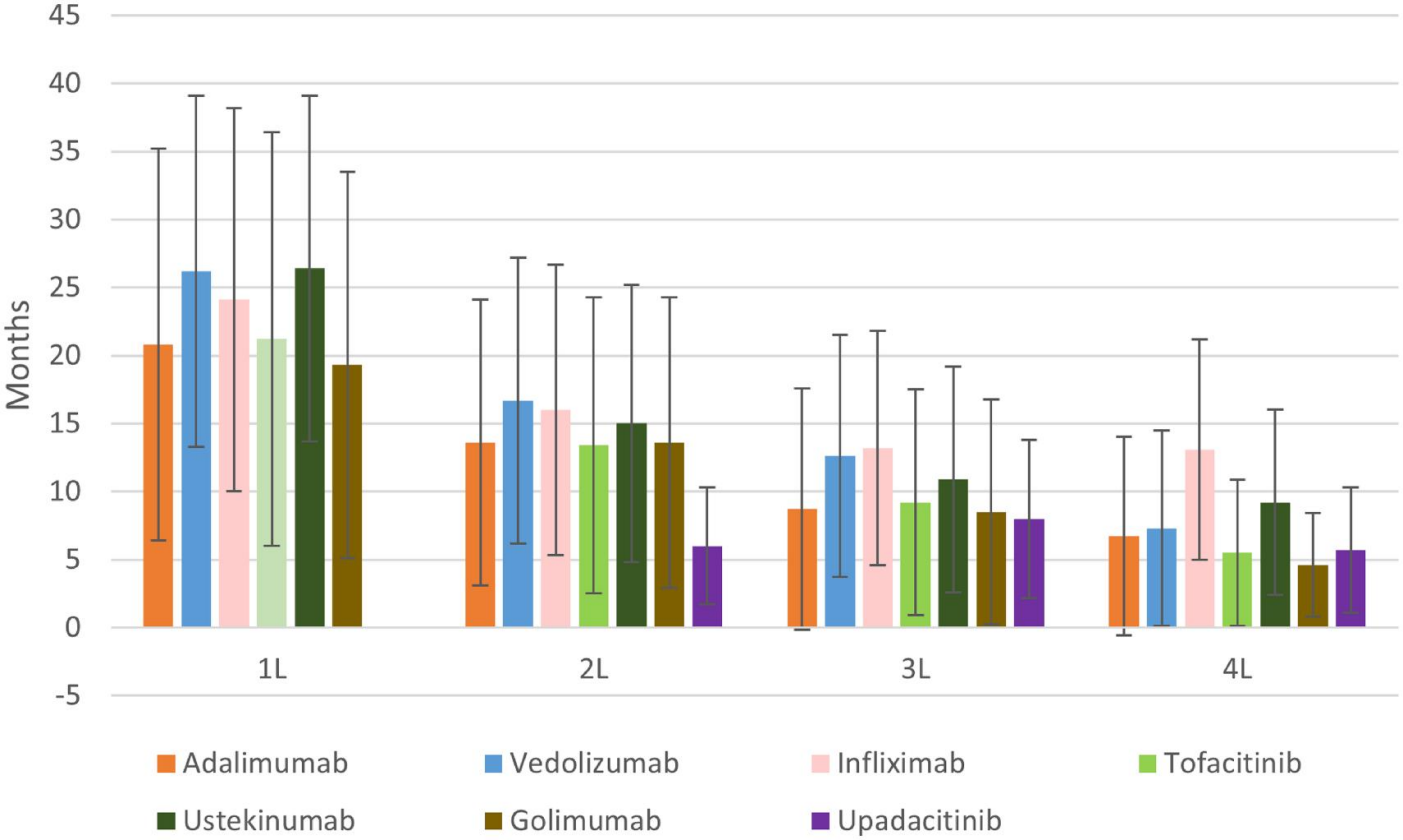
Duration of treatment. *Notes*: (1) Duration of treatment was defined as the time from index date to the last day of supply of the last claim for the advanced therapy. (2) The bars represent the mean duration of treatment (in months) for each therapy across different lines of treatment (1L, 2L, 3L, 4L). Error bars indicate the standard deviation (SD), reflecting variability in treatment duration among patients.

### Dose escalation

Around 27%-31% of patients underwent a dose escalation at some point in their treatment line (Appendix Table S5). Across all lines of therapy, patients on tofacitinib and ustekinumab experienced the highest rates of dose escalation, with 31.7% to 51.7% of patients on tofacitinib and 31.2% to 61.0% of patients on ustekinumab experiencing dose escalation across 1L to 4L (Figure 3B).

### Switching

Among the 6726 patients who initiated an AT, 39.6% switched to a different AT at some point during the follow-up period (Appendix Table S5). The switching rates generally decreased in subsequent line of therapies (30.5% in 2L; 24.9% in 3L; 33.7% in 4L). Among patients who switched, 41.5%, 43.3%, 45.0%, and 51.5% were on GC at the time of switching in the 1, 2, 3, and 4L therapy, respectively. Figure 3C presents the switching patterns by individual treatment and lines of therapy.

### Restarting

A minority of patients restarted AT after a substantial gap in treatment, with 9.0%, 5.5%, and 6.2% of the overall population restarting the same AT in the 1, 2, and 3L of therapy, respectively. The restarting rate was the highest among patients using tofacitinib across 1L to 4L (Figure 3D).

## Discussion

This study provides long-term (3+ years), real-world insights into treatment patterns with ATs among patients with UC in the United States. Our study results suggest that ATs for UC are associated with high rates of discontinuation, switching, and need for dose escalation or use of steroids. A clear gap remains in the field for durable treatment options to induce and maintain steroid-free disease remission.

High rates of switching and discontinuation observed in our study are consistent with previous studies investigating treatment patterns among UC patients in the U.S. using administrative claims data. Lee and colleagues reported that 38% of patients switched to a different AT and 26% discontinued the AT without starting a new AT.^10^ Sah and colleagues observed discontinuation without restart or switching rates ranged from 14.4% to 26.7% across included medications in the first year of treatment.^14^ Another study reported a lower switching rate of 16% though it used a more restrictive definition; patients needed to switch to another AT within 90 days following the last day of supply of the initial AT.^15^ The higher discontinuation and switching rates in our study likely reflect the longer follow-up period (mean: 61.5 months), given complete discontinuation rates were much higher when patients were followed for more than two years.

The high rate of complete discontinuation warrants further investigation, as it is unlikely to be explained by surgery or loss to follow-up, given patients were observed for at least one year after discontinuation and colectomy rates in the US for UC are less than 10% and declining over time after the introduction of newer AT agents.^16^^,^^17^ One possible explanation is that some patients may face financial barriers, such as high co-payment requirements, that discourage continued use of ATs. Alternatively, some patients may achieve disease remission and choose to trial stopping the AT to avoid long-term use of drugs that suppress the immune system. This may be reflected in part by the observed frequency of restarting AT in our dataset. Traditional monoclonal antibodies have limitations when being used in an interrupted fashion due to the development of anti-drug antibodies and we observed restarting of monoclonal antibodies to be less than 10%. Oral small molecule agents do not carry this risk, and we observed oral small molecules (JAK inhibitors) were more often restarted (>30% in first line use).

Our study results indicate that approximately 27%-31% of patients experienced a dose escalation at some point in their treatment line, largely in agreement with a previous study of US based claims data from 2005-2013 which estimated dose escalation rates to be 44% within 36 months of index AT initiation.^15^ That study was done prior to the approval of vedolizumab, ustekinumab, or JAK inhibitors, and our results help expand these estimates to current treatment landscapes. Furthermore, we expand on prior persistence estimates by incorporating a measure accounting for both dose escalation and GC use. The combined use of both measures helped better define the low rate of treatment persistence in practice over time. A clear gap remains for treatment options in moderate-severe UC that can achieve durable steroid-free disease remission at FDA labeled doses.

The study has several limitations. First, administrative claims provide indirect evidence of adherence, and we cannot observe reasons for discontinuations, switching, and restarting outside of claims. Second, medical claims do not have adequate information to definitively identify dosage for IV drugs. Our definition for dose escalation required observing a maintenance dose twice as high as the FDA-recommended standard maintenance dose for UC or interval shortening, which may not always capture real-world clinical practice, where physicians may increase dose to achieve target plasma concentration for patients, rather than strictly doubling the dose. This approach was conservative and may lead to underestimates of dose escalations for infliximab in particular. Third, certain lifestyle factors that can influence disease and treatment outcomes, such as smoking, are underreported in claims data and their impact on the study outcomes could not be ascertained. Fourth, the study design required patients to have at least 36 months of continuous health insurance enrollment following initiation of AT. To the degree that loss to follow up is associated with loss of insurance coverage (rather than, for example, job switching), our findings may overstate treatment persistence. Lastly, since the study population was commercially insured, the study results may not be generalizable to the US population with non-commercial insurance types, such as traditional Medicare and Medicaid, or to uninsured people or people outside the US.

The treatment landscape for moderate-to-severe UC has evolved with the introduction of new ATs, but treatment patterns have not evolved and a substantial proportion of patients either require dose optimization, continued steroid use, or switching of therapies. The high rate of AT discontinuation further demonstrates a clear need to identify novel oral therapeutic targets, including potential combination therapies, that can achieve long-term durable steroid-free disease remission and be restarted if treatment interruption does occur.

## Supplementary Material

otag035_Supplementary_Data

## Data Availability

No public data available.
