# Supplementary material for: Advanced therapy treatment patterns in moderate-to-severe ulcerative colitis: a long-term retrospective claims analysis
Source: Crohns Colitis 360. 2026 Apr 22;8(2):otag035. doi: 10.1093/crocol/otag035 (PMC13220750; doi:10.1093/crocol/otag035)
Supplement: otag035_Supplementary_Data [file otag035_supplementary_data.zip › 2025.07.10 UCTX Supplement_Submitted to CC360.docx]

# Supplementary Appendix

## Appendix Table 1. Diagnosis Codes for Ulcerative Colitis and Other Autoimmune Diseases

| **Condition** | **ICD-9 Codes** | **ICD-10 Codes** |
| --- | --- | --- |
| Ulcerative Colitis (UC) | 556 | K51 |
| Multiple Sclerosis (MS) | 340 | G35 |
| Rheumatoid Arthritis (RA) | 714 | M05, M06, M12 |
| Psoriasis | 6961, 6968 | L400, L401, L402, L403, L404, L408, L409 |
| Psoriatic Arthritis | 6960 | L405 |
| Ankylosing Spondylitis | 720 | M45, M488 |
| Uveitis | 36400, 36401, 36402, 36404, 36405, 36410, 36411, 36421, 36422, 36423, 3643, 36321, 36012, 36212, 36218, 36300, 36301, 36303, 36304, 36305, 36306, 36310, 36311, 36312, 36313, 36315, 36320, 36322, 36424 | H2000, H2001, H2002, H2004, H2005, H201, H202, H209, H2081, H2082, H3000, H3001, H3002, H3003, H3004, H302, H309, H3010, H3011, H3012, H3013, H3014, H3081, H3089, H3502, H3506, H4040, H4411 |
| Hidradenitis Suppurativa | 70583 | L732 |
| Autoimmune Hepatitis | 57142 | K754 |
| Malignancies | 14, 15, 16, 17, 18, 19, 20 | C |
| Crohn's Disease (CD) | 555 | K50 |
| Indeterminate Colitis (IC) | 5589 | K523 |

## Appendix Table 2. Codes for Ulcerative Colitis Advanced Therapies

| **Therapy** | **HCPCS Codes** | **NDC Codes** | **GPI Codes** |
| --- | --- | --- | --- |
| Infliximab | J1745, Q5102, Q5103, Q5104, Q5109, Q5121 | 57894003001, 00069080901, 00006430502 |  |
| Adalimumab | J0135 | 00074379902, 00074433906, 00074433902, 00074433907, 00074937402, 00074634702, 00074024302, 00074055402, 00074061602, 00074081702, 00074012403, 00074153903, 00074379901, 00074379903, 00074379906, 54868482200, 54569552400 |  |
| Vedolizumab | C9026, J3380 | 64764030020 |  |
| Ustekinumab | J3357, J3358, C9261, C9487, Q9989 | 57894005427, 57894006003, 57894006103, 57894006002 |  |
| Golimumab | J1602 | 57894007101, 57894007102, 57894007001, 57894007002, 57894035001 |  |
| Tofacitinib |  | 00069100201, 00069050230, 00069050130, 00069100101 |  |
| Upadacitinib |  |  | 6660307200 |

## Appendix Table 3. Dose Escalation Thresholds for UC Advanced Therapies

| **Advanced Therapy** | **Recommended Maintenance Interval** | **Recommended Maintenance Dose** | **Recommended Maintenance Daily Dose** | **Thresholds for  Dose Escalation** |
| --- | --- | --- | --- | --- |
| Adalimumab | Every other week | 40 mg | 40mg/14 days | Daily dosage > 2*(40mg/14 days) |
| Infliximab | Every 8 weeks | - | - | Interval shortening (≤6 weeks) |
| Vedolizumab | Every 8 weeks | - | - | Interval shortening (≤6 weeks) |
| Ustekinumab | Every 8 weeks | 90 mg | 90 mg/56 days | -Double the dosage, or -Interval shortening (≤6 weeks), or -Infusion (medical) -> injection (pharmacy) -> infusion (medical) |
| Tofacitinib | 5 mg twice daily or 11 mg once daily | 5 mg or 11 mg | 10 mg or 11 mg | Daily dosage > 2*(10 mg/day) |
| Golimumab | Every 4 weeks | 100 mg | 100 mg/28 days | -Daily dosage > 2*(100 mg/28 days), or  Interval shortening (≤2 weeks) |
| Upadacitinib | Once daily | 15 mg | 15 mg/day | Daily dosage > 2*(15 mg/day) |

## Appendix Table 4. Patient Baseline Characteristics by Index Year

|  | **2012-2014** | **2015-2016** | **2017-2018** | **2019-2020** |
| --- | --- | --- | --- | --- |
|  | **N = 1,096** | **N = 1,542** | **N = 1,900** | **N = 2,188** |
| **Demographics** |  |  |  |  |
| Age at index (years) |  |  |  |  |
| Mean ± SD | 39.4 ± 14.0 | 38.7 ± 14.2 | 39.1 ± 14.6 | 38.6 ± 14.9 |
| Sex |  |  |  |  |
| Female | 496 (45.3%) | 731 (47.4%) | 909 (47.8%) | 1,006 (46.0%) |
| Male | 600 (54.7%) | 811 (52.6%) | 991 (52.2%) | 1,182 (54.0%) |
| **Clinical characteristics** |  |  |  |  |
| Time from first observed UC diagnosis to index date (month) | |  |  |  |
| Mean ± SD | 3.8 ± 1.7 | 3.8 ± 1.8 | 3.5 ± 1.7 | 3.5 ± 1.7 |
| **Charlson Comorbidity Index, n (%)** |  |  |  |  |
| CCI composite score |  |  |  |  |
| Mean ± SD | 0.2 ± 0.7 | 0.3 ± 0.7 | 0.2 ± 0.6 | 0.2 ± 0.6 |
| Chronic pulmonary disease | 92 (8.4%) | 123 (8.0%) | 167 (8.8%) | 168 (7.7%) |
| Mild liver disease | 62 (5.7%) | 88 (5.7%) | 85 (4.5%) | 111 (5.1%) |
| Diabetes without chronic complications | 58 (5.3%) | 81 (5.3%) | 93 (4.9%) | 97 (4.4%) |
| Peripheral vascular disease | 14 (1.3%) | 21 (1.4%) | 31 (1.6%) | 34 (1.6%) |
| Renal disease | 10 (0.9%) | 27 (1.8%) | 13 (0.7%) | 29 (1.3%) |
| Peptic ulcer disease | 9 (0.8%) | 21 (1.4%) | 24 (1.3%) | 24 (1.1%) |
| **Comorbidities related to UC, n (%)** |  |  |  |  |
| Diarrhea | 592 (54.0%) | 779 (50.5%) | 951 (50.1%) | 1,073 (49.0%) |
| Anemia | 154 (14.1%) | 266 (17.3%) | 324 (17.1%) | 364 (16.6%) |
| Hypertension | 169 (15.4%) | 222 (14.4%) | 285 (15.0%) | 333 (15.2%) |
| Obesity | 87 (7.9%) | 191 (12.4%) | 295 (15.5%) | 371 (17.0%) |
| Anxiety | 103 (9.4%) | 182 (11.8%) | 259 (13.6%) | 340 (15.5%) |
| Fatigue | 147 (13.4%) | 199 (12.9%) | 244 (12.8%) | 246 (11.2%) |
| Depression | 100 (9.1%) | 161 (10.4%) | 181 (9.5%) | 256 (11.7%) |
| Infections | 64 (5.8%) | 98 (6.4%) | 145 (7.6%) | 125 (5.7%) |
| Malnutrition | 48 (4.4%) | 50 (3.2%) | 93 (4.9%) | 102 (4.7%) |
| **Prior UC treatments, n (%)** |  |  |  |  |
| Any prior use of UC treatments | 1,013 (92.4%) | 1,430 (92.7%) | 1,730 (91.1%) | 2,019 (92.3%) |
| **Glucocorticoids** |  |  |  |  |
| Any prior use of glucocorticoids | 916 (83.6%) | 1,261 (81.8%) | 1,491 (78.5%) | 1,708 (78.1%) |
| Prednisone | 788 (71.9%) | 1,057 (68.5%) | 1,243 (65.4%) | 1,417 (64.8%) |
| Budesonide | 271 (24.7%) | 428 (27.8%) | 454 (23.9%) | 521 (23.8%) |
| Hydrocortisone | 197 (18.0%) | 237 (15.4%) | 251 (13.2%) | 236 (10.8%) |
| Methylprednisolone | 121 (11.0%) | 162 (10.5%) | 201 (10.6%) | 222 (10.1%) |
| **Oral 5-ASA** |  |  |  |  |
| Any prior use of oral 5-ASA | 855 (78.0%) | 1,162 (75.4%) | 1,408 (74.1%) | 1,620 (74.0%) |
| Mesalamine | 775 (70.7%) | 1,062 (68.9%) | 1,264 (66.5%) | 1,489 (68.1%) |
| Balsalazide | 101 (9.2%) | 109 (7.1%) | 151 (7.9%) | 146 (6.7%) |
| Sulfasalazine | 54 (4.9%) | 78 (5.1%) | 100 (5.3%) | 110 (5.0%) |
| **Immunomodulators** |  |  |  |  |
| Any prior use of immunomodulators | 273 (24.9%) | 328 (21.3%) | 330 (17.4%) | 289 (13.2%) |
| Azathioprine | 254 (23.2%) | 290 (18.8%) | 274 (14.4%) | 246 (11.2%) |
| Mercaptopurine | 145 (13.2%) | 174 (11.3%) | 147 (7.7%) | 113 (5.2%) |
| Methotrexate | 17 (1.6%) | 29 (1.9%) | 44 (2.3%) | 31 (1.4%) |

**Abbreviations**: 5-ASA, 5-aminosalycylic acid; CCI, Charlson Comorbidity Index; HIV/AIDS, human immunodeficiency virus/acquired immunodeficiency syndrome; SD, standard deviation; UC, ulcerative colitis.

**Notes**:
[1] The index date was defined as the date of treatment initiation for a patient’s first treatment for moderate to severe UC.
[2] The baseline period was defined as the 6 months prior to the index date.
[3] The UC diagnosis was observed during the baseline period.
[4] The IQVIA data provides only the year of birth (YOB), so July 1st of the birth year was used to impute the date of birth (DOB). Age at the index date was then calculated as the difference between the index date and the imputed DOB.
[5] Some conditions may be under-reported in claims and therefore prevalence of UC-related comorbidities may be underestimated.
[6] For comorbidities included in the CCI, a total of 0 (0.0%) patients had claims with a diagnosis of any malignancy, including lymphoma and leukemia, except malignant neoplasm of skin, or metastatic solid tumor.
[7] For UC-related comorbidities, a total of 0 (0.0%) patients had claims with a diagnosis of smoking.
[8] A total of 0 (0.0%) patients had claims for treatment with Beclomethasone, Sirolimus, Thalidomide, or Thioguanine during the baseline period. [9] Variables with cell sizes < 11 were suppressed to protect privacy and maintain compliance with data reporting guidelines. The comorbidities suppressed include congestive heart failure, diabetes with chronic complications, myocardial infarction, cerebrovascular disease, rheumatic disease, moderate to severe liver disease, hemiplegia or paraplegia, HIV/AIDS, and dementia. Prior UC treatments suppressed include prednisolone, dexamethasone, olsalazine, tacrolimus, mycophenolate, and cyclosporine.

## Appendix Table 5. Treatment Failure Events Among the Overall Population by Line of Treatment

|  | **1L** | **2L** | **3L** | **4L** |
| --- | --- | --- | --- | --- |
|  | **N = 6,726** | **N = 2,663** | **N = 812** | **N = 202** |
| **Follow-up time (months)^1^** |  |  |  |  |
| Mean ± SD | 61.5 ± 21.3 |  |  |  |
| Median | 55.9 |  |  |  |
| IQR | (44.3, 73.7) |  |  |  |
| **Number of patients with only 1 claim of index advanced therapy** | 609 (9.1%) | 345 (13.0%) | 146 (18.0%) | 50 (24.8%) |
| **Number of patients with dose escalation events** | 1,818 (27.0%) | 753 (28.3%) | 249 (30.7%) | 56 (27.7%) |
| **Number of patients who switched to a different advanced therapy, n (%)** | 2,663 (39.6%) | 812 (30.5%) | 202 (24.9%) | 68 (33.7%) |
| Had a claim for their index therapy after switch, n (%) | 129 (4.8%) | 25 (3.1%) | 8 (4.0%) | 0 (0.0%) |
|  |  |  |  |  |
| On glucocorticoids at switching, n (%) | 1,105 (41.5%) | 352 (43.3%) | 91 (45.0%) | 35 (51.5%) |
| On prednisone at switching, n (%) | 914 (34.3%) | 295 (36.3%) | 80 (39.6%) | 31 (45.6%) |
| On budesonide at switching, n (%) | 248 (9.3%) | 76 (9.4%) | 21 (10.4%) | 8 (11.8%) |
| **Number of patients who restarted index advanced therapy, n (%)** | 602 (9.0%) | 147 (5.5%) | 50 (6.2%) | 7 (3.5%) |
| **Number of patients with a complete discontinuation event, n (%)** | 3,747 (55.7%) | 1,704 (64.0%) | 552 (68.0%) | 128 (63.4%) |
| With a delayed censoring event, n (%) | 780 (20.8%) | 398 (23.4%) | 141 (25.5%) | 41 (32.0%) |
| With at least one year between discontinuation date and end of | 2,967 (79.2%) | 1,306 (76.6%) | 411 (74.5%) | 87 (68.0%) |
| follow-up, n (%) |  |  |  |  |
| On glucocorticoids at the time or after time of discontinuation, n (%) | 1,608 (42.9%) | 853 (50.1%) | 315 (57.1%) | 66 (51.6%) |
| On glucocorticoids at the time or within 3 months of discontinuation,    n (%) | 540 (13.3%) | 349 (18.9%) | 158 (25.9%) | 40 (29.9%) |
| **Duration of treatment (months)** |  |  |  |  |
| Mean ± SD | 22.9 ± 14.2 | 15.5 ± 10.6 | 11.2 ± 8.7 | 7.8 ± 6.9 |
| Median | 25.8 | 13.7 | 8.9 | 5.2 |
| IQR | (7.9, 36.5) | (5.8, 25.0) | (3.2, 17.7) | (1.8, 12.5) |

Note:
[1] Follow-up time (months) was summarized as the total follow-up time in the overall population (N=6,726) regardless of line of treatment.

## Appendix Figure 1. Study Population Flow Chart

**Abbreviations**: ICD-09-CM: International Classification of Diseases 9th Revision, Clinical Modification; ICD-10-CM: International Classification of Diseases 10th Revision, Clinical Modification; UC: Ulcerative Colitis.

**Note**: Other diseases include rheumatoid arthritis, psoriasis, psoriatic arthritis, ankylosing spondylitis, noninfectious uveitis, hidradenitis suppurativa, and any type of malignancy.
